# The Impact of an Outdoor Motor–Cognitive Exercise Programme on the Health Outcomes of Older Adults in Community Settings: A Pilot and Feasibility Study

**DOI:** 10.3390/sports12020049

**Published:** 2024-02-01

**Authors:** Katharina Zwingmann, Torsten Schlesinger, Katrin Müller

**Affiliations:** Institute of Human Movement Science and Health, Faculty of Behavioral and Social Sciences, Chemnitz University of Technology, 09107 Chemnitz, Germany; torsten.schlesinger@hsw.tu-chemnitz.de (T.S.); katrin.mueller@hsw.tu-chemnitz.de (K.M.)

**Keywords:** community health promotion, older adults, physical activity, intervention, outdoor, physical capacity, cognitive health

## Abstract

Physical and cognitive exercises can prevent or at least mitigate the symptoms of certain diseases and help older adults perform a range of daily functions. Yet, most seniors do not meet the World Health Organisation’s recommended guidelines for physical activity. The objective of this study is to promote and maintain the physical and cognitive capacity of older adults by implementing a feasible and effective low-threshold, age-appropriate, motor–cognitive training outdoors. In the German city of Chemnitz, citizens aged 60 years and older participated in a quasi-randomised intervention trial. Exercises to train coordination, strength, endurance, and cognition were integrated into a 12-week outdoor motor–cognitive exercise programme. Both the physical (e.g., 6MWT) and cognitive skills (e.g., TMT B) of the intervention group (*n* = 41) and control group (no intervention, *n* = 58) were measured before (T1) and after (T2) completion of the exercise programme. Some of the participants’ physical and all their cognitive measures improved. Neurocognitive performance (DSST) showed a significant time × group interaction effect (F(1,95) = 6.943, *p* = 0.010, $\eta_{p}^{2}$ = 0.068). Sex and age were found to be influencing factors. We consider our exercise programme to be successfully implemented, well received by the participants, and feasible and useful to promote the continued exercise of daily functions as part of healthy aging in community-dwelling older adults.

## 1. Introduction

Despite rising migration, demographic change will continue and intensify further in countries such as Germany [1]. The German Federal State of Saxony is disproportionately affected by an increasing ageing population: in 2018, the average age of Saxony’s population was 46.8 years, with 26.1% aged 65 years and older. A further increase is anticipated due to growing life expectancy and falling birth rates [2]. Ageing societies pose a major challenge for healthcare systems due to the rise in chronic and neurodegenerative diseases related to the process of ageing [3], resulting in an upsurge in total healthcare costs [4]. Demographic management in ageing societies requires special efforts to foster healthy ageing; these efforts, among others, include the promotion of good health as well as the maintenance of autonomy and well-being in old age [5].

### 1.1. Promoting and Maintaining Health in Older Age through Physical Activity

Maintaining an active lifestyle through regular physical activity helps older adults (OAs) to ensure their ability to engage fully in society, preserves their functional independence, health, and well-being, and thus supports healthy ageing [6]. Several recent studies indicate the positive effects of regular sport and physical activity on OAs’ physical, psychosocial, and cognitive health [7,8,9,10]. For example, participation in physical activity can be beneficial in maintaining (self-rated) health and physical function, preserving mobility, and reducing fall-related injuries, thereby contributing to autonomous and healthy ageing and a higher quality of life [11,12,13]. The positive effects of regular physical activity are evident in several health-related dimensions, such as a lower risk of mortality [7,14] and cardiovascular diseases [15,16], delayed decline in cognitive function [17,18] and a beneficial influence on (mental) health and well-being [19,20,21].

Additional positive effects at both the cognitive (e.g., attention span) [22] as well as physical level (e.g., balance) [23] can be achieved through dual task training, i.e., combining cognitive and motor activity in exercises. Most coordination exercises (e.g., dancing, step aerobics) directly link a cognitive task to the simultaneous performance of a motor task [24]. Training motor–cognitive dual tasks is relevant for OAs’ activities of daily living, such as mobility, household chores, or social interactions, and can help maintain independence [25]. Including coordination exercises in physical activity programmes for OAs has more beneficial effects on cognitive performance than strength or endurance training alone [26]. A meta-analysis carried out by Gheysen et al. [27] finds that physical activity programmes that include more demanding cognitive challenges have the potential to improve OAs’ cognitive health and function. General cognitive function and inhibitory control can be improved through loaded cognitive-motor training, which involves dual-task exercises with progressive difficulty [28]. Compared to multi-component training, which focuses on physical performance, executing certain functions seems to be further enhanced through concurrent cognitive-physical training [29].

Moreover, physical activity, particularly when performed outdoors, has additional positive effects on health: improved interpersonal relationships, reduced sense of exertion, increased revitalisation, happiness, and satisfaction, and a decrease in psychological symptoms, e.g., depression [30]. Furthermore, outdoor living environments can indirectly positively affect biopsychosocial health by influencing health-promoting behaviour (e.g., green space as an incentive for exercise) [31]. Urban green space can be seen as a design element of socially equitable urban development, provided it is accessible and usable for everyone. Therefore, when practicing physical activity outdoors, green space represents an opportunity structure and a motivational factor [32,33].

Studies show that outdoor physical activity has moderately positive effects on a person’s overall sense of well-being [34]. However, the most relevant advantage of outdoor versus indoor physical activity programmes is the greater willingness of participants to remain physically active over the long term [35,36]. That is, if physical activity takes place outdoors, it can, over time, lead to more health-promoting behaviour among OA.

### 1.2. Influencing Factors and Suitable Settings for Implementing Regular Physical Activity in Old Age

Although OAs can significantly benefit from participation in physical activity, the proportion of those in Germany who meet the World Health Organisation’s (WHO) [37] recommended guidelines for physical activity is low [38,39]. The share of OAs who are physically active lies far below the German population’s average [2]. The figure is even lower among socio-economically disadvantaged groups, e.g., females, people with lower levels of education, socially isolated persons, and people with a low income [40,41,42]. Vulnerability in older age intensifies functional limitations, which in turn makes day-to-day activities more difficult and is more likely to lead to a permanent need for care [43]. A large proportion of OAs are thus not able to engage in adequate physical activity, and consequently, they only disproportionately benefit from its health effects. Access for OAs to health promotion measures is associated with higher barriers [44]. Environmental factors and a lack of resources are the most common barriers to health promotion interventions among OA [45]. Motivators to engage in physical activity, on the other hand, include improving one’s physical condition, addressing psychological issues, socialising, supervision by health professionals, as well as the suitability and safety of the physical environment [46].

### 1.3. Reflection and Consequences

Target group-specific, efficient physical activity-stimulating programmes for OA are particularly beneficial. Previous health-promoting programmes that have intended to increase the level of regular physical activity among OA have mostly achieved only limited success [47,48]. One possible reason is that such measures have previously not been adequately adapted to OAs’ individual conditions and needs, have been associated with very high financial costs, were planned with high thresholds, or took place far from their place of residence [49,50,51].

There is often a lack of low-threshold and barrier-free mobility offers in OAs’ direct living environments. In terms of accessibility, a demand-oriented and group-based programme delivered within close proximity of the place of residence that incorporates the existing setting and structures of community health promotion is suitable [42]. OAs spend a large part of their day in their immediate living environment. Accordingly, the built environment in their vicinity might influence their engagement in physical activity, e.g., accessibility to public open spaces and green areas as well as infrastructure and services [52]. Local structures must be taken into consideration when aiming to reach vulnerable, previously inactive population groups, such as OAs, with the purpose of promoting physical activity [53,54].

To sum up, in the literature studies about the effects of physical exercise on balance and fall risk of community-dwelling individuals, e.g., [11], or the effect of a physical exercise multicomponent training with cognitive elements, e.g., [29], can be found. But reviews and meta-analyses investigating the effects of physical activity as group interventions are rare [55], especially for the target group of OAs in combination with the execution in the community outdoor setting. In German culture, the use of neighbourhood outdoor settings for (group) exercises is not (yet) a common practice, particularly within OAs. To bridge this gap, the objective of our study was to examine the feasibility and effectiveness of a low-threshold age-appropriate outdoor motor–cognitive training for OAs to promote and maintain the physical and cognitive capacity of this target group in the long term. Hence, a needs-specific, group-based, and motoric-cognitive exercise intervention for OAs was conceptualised and implemented, which is firstly free of charge, secondly can be continued independently after the end of the study period, and, thirdly, positive effects and feasibility assumed, would be transferable to other municipalities. This study addresses the following research-guiding questions:

- What impact does a 12-week outdoor motor–cognitive exercise programme have on OAs’ physical and cognitive function?
- Are there any age- and sex-related differences in terms of the motor–cognitive exercise programme’s impact?
- How is the feasibility of and satisfaction with the outdoor motor–cognitive exercise programme within the target group?

## 2. Methodological Approach

The reporting of this trial is based on the guidelines of the CONSORT statement [56].

### 2.1. Trial Design

**Figure 1:**
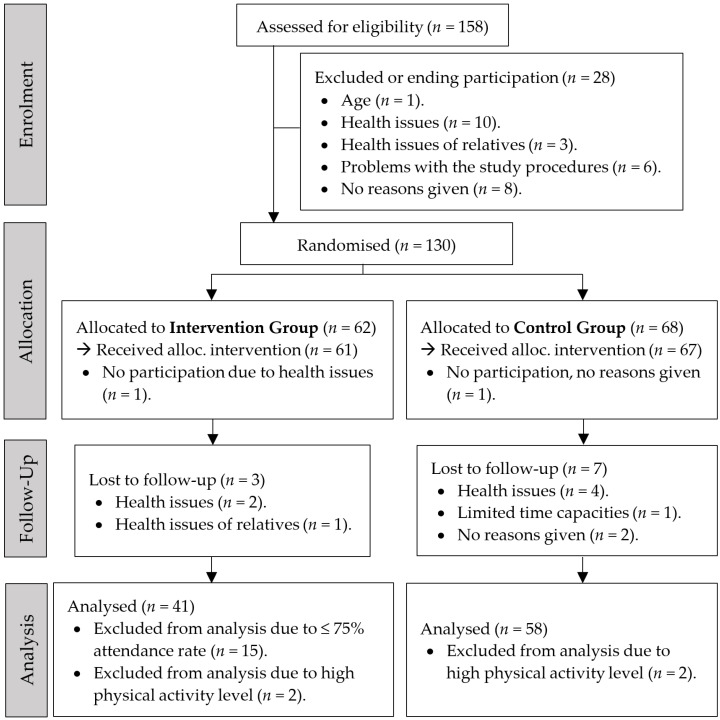
Study procedure of enrolment, allocation, follow-up, and analysis of participants (intervention and control group).

The present study was conducted as a two-arm quasi-randomised controlled trial to investigate the feasibility and effects of a motor–cognitive exercise programme in the outdoor living environment within a pilot setting for OAs. An overview of the study’s procedure is presented in Figure 1. The trial was registered at the German Register for Clinical Studies (“Deutsches Register für Klinische Studien”/DRKS), registration ID number DRKS00032156.

### 2.2. Sample Size Estimate/Power Calculations

Due to the pilot character of the study design, which sought to test the feasibility and impact of the motor–cognitive exercise programme, a post hoc power analysis was conducted using G*Power (Version 3.1.9.7, Heinrich-Heine-University Düsseldorf) [57]. For the statistical test of repeated measures, ANCOVA with an effect size f(V) of 0.25, an α of 0.05, a total sample size of 99, a numerator df of 1, 2 groups, and 2 covariates were used. The post hoc analysis calculated a non-centrally parameter λ of 6.188, a critical F-value of 3.941, a denominator df of 95, and a power (1-β) of 0.692.

### 2.3. Study Setting and Participants

Older citizens of the German city of Chemnitz in the federal state of Saxony (population: 249,458, of which 34.4% are people aged 60 years or older; 31 January 2023; data from the City Council) were invited to participate in the study and the first measurement date. Participants were recruited through flyers, personal contacts at community centres, advertisements in a local newspaper, and previous participant lists of research projects. The inclusion criteria for participation in the study were that the individual had to be 60 years or older and a resident of the City of Chemnitz. Individuals with a medical prohibition of physical activity, those who had experienced critical health events over the last six months (e.g., myocardial infarction, stroke), had been diagnosed with dementia or major depression, or who were already participating in other clinical studies, were excluded. Figure 1 presents the study’s procedure for enrolment, allocation, follow-up, and analysis of participants.

### 2.4. Randomisation and Assignment to Intervention Group

The city districts of the registered participants were ranked according to socio-economic status (evaluated using an overall index based on population density, out-migration/in-migration, housing, assistance receipt, or households of the district residents, among others) [58]. Three city districts (from thirty-nine) were selected for participants in the intervention group (IG), while four socio-economically comparable city districts were identified for the control group (CG). Using this approach, the number of enrolled participants was evenly distributed between the two groups. The IG (*n* = 62) was further divided into five exercise groups based on the number of participants living in each city district. Hence, two exercise groups were created for the city’s central east district (IG1, *n* = 11; IG2, *n* = 11), two for the south district (IG3, *n* = 16; IG4, *n* = 13), and one for the west district (IG5, *n* = 11). The CG consisted of 68 participants.

The rate of attendance for the IG to be included in the analysis was set at 75%, i.e., participants had to attend 18 out of the 24 units of the intervention. A total of 15 IG participants did not fulfil this rate of attendance and were therefore excluded from the analysis. Participants who already followed the national and international exercise recommendations of 150 min of moderate physical activity per week [37,39] were also excluded from the analysis due to their high level of regularly physical activity. To determine compliance with the WHO’s recommendations for physical activity, one question from the Freiburg Physical Activity Questionnaire (“Freiburger Fragebogen für körperliche Aktivität/FFkA“) [59] was used (“How often do you exercise?”; answer options: “no sports activities”/“less than 1 h per week”/“regularly, 1–2 h per week”/“regularly, 2–4 h per week”/“regularly, more than 4 h per week”; cut-off between “regularly, 2–4 h per week” and “regularly, more than 4 h per week”). Two participants from each group were excluded from the analysis based on this cut-off value. In total, the data of 99 participants were analysed, namely 41 IG participants and 58 CG participants.

### 2.5. Characteristics of Participants

Table 1 presents the participants’ characteristics, separated for the two groups. The Montreal Cognitive Assessment’s (MoCA) [60] short screening instrument was used to control for equal balance between the two groups in terms of general cognitive health. By categorising the MoCA’s total score, the participants were classified as cognitively healthy (a score between 30 and 26 points), mildly cognitively impaired (a score between 25 and 19 points), or severely cognitively impaired (a score of 18 or lower). In the IG, 61.0% of participants were classified as cognitively healthy, while 39.0% showed mild cognitive impairment. Among the members of the CG, 74.1% were cognitively healthy, and 25.9% were categorised as mildly cognitively impaired.

IG and CG differed significantly in age and general cognitive health (MoCA), but not in body mass index (BMI), comorbidities (ACCI; measured by age-adjusted Charlson Comorbidity Index) [61], or subjective socio-economic status (SSES; measured using the German version of the MacArthur scale with steps from 1 to 10) [62]. The sex distribution between both groups was balanced, with 60% female participants and no significant difference (F(1,98) = 0.012, *p* = 0.913, $\eta_{p}^{2}$ = 0.000). Regarding age, within the IG (women mean age: 72.48 ± 6.15 years; men mean age: 72.44 ± 6.52 years; F(1,40) = 0.000, *p* = 0.983, $\eta_{p}^{2}$ = 0.000) as well as within the CG (women mean age: 74.47 ± 7.95 years; men mean age: 77.55 ± 7.10 years; F(1,57) = 2.207, *p* = 0.143, $\eta_{p}^{2}$ = 0.038), there were no significant sex differences. The sex distribution in this study corresponds approximately to that in the City of Chemnitz (surplus of women with 56.9% in 2021; data from the City Council). Both groups fell within the optimum range of BMI levels for OAs [63]. The FFkA question provided the following values for the current amount of physical activity in the sample: 19.5% of IG and 12.3% of CG performed no sports activities; 24.4% of IG and 12.3% of CG were physically active less than 1 h per week; 39.0% of IG and 56.1% of CG were regularly physically active with 1–2 h per week; 17.1% of IG and 19.3% of CG were regularly physically active with 2–4 h per week.

### 2.6. Design and Procedure of Intervention

**Figure A1:**
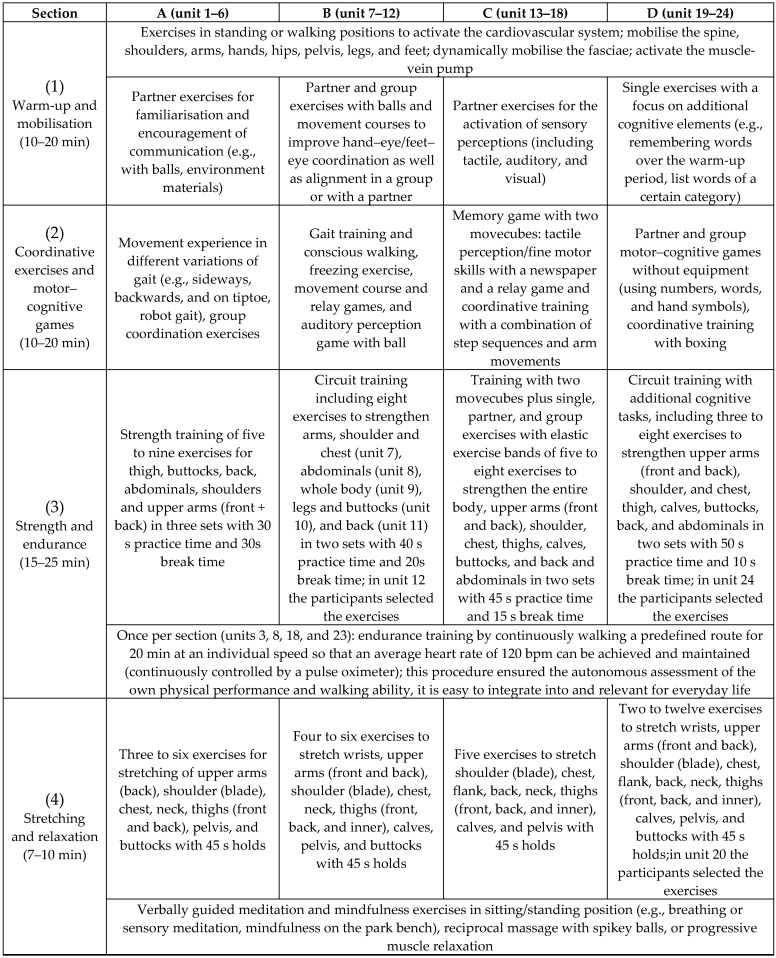
Description of the structure (1) to (4) of the four sections (A–D) of the outdoor motor–cognitive exercise programme.

**Figure A2:**
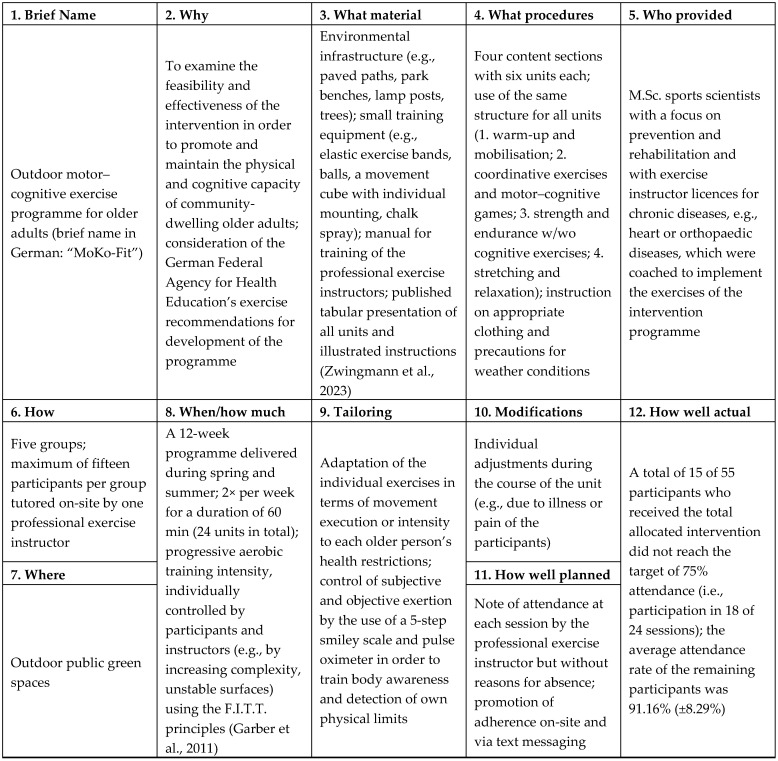
Complete checklist of the template for intervention description and replication (TIDieR) [64] for our exercise programme [65,67].

The description of the intervention is based on the Template for Intervention Description and Replication (TIDieR) Checklist and Guide [64]. Zwingmann, Schlesinger, and Müller (in German) [65] provided a comprehensive tabular presentation of all units of the outdoor motor–cognitive exercise programme as well as illustrated instructions for individual exercises. Figure A1 summarises the content of the individual units (content sections, structure, and sequence), including examples of exercises. Figure A2 provides the complete TIDieR Checklist.

To achieve optimal health benefits among participating OAs, their main motor functions (strength, endurance, coordination, and mobility) as well as their everyday cognitive functions (e.g., memory, attention, and mental flexibility) were trained by combining both physical and cognitive tasks in the different exercises [25]. The German Federal Agency for Health Education’s exercise recommendations for OAs [66] were considered when developing this programme. The intervention comprised a total of 24 units carried out over a 12-week period (2×/week for 60 min). Four content sections of six units each were planned, consisting of repetitive exercises with increasing intensity and a set of different goals (see Figure A1 for a detailed description of the different sections). For all the exercise programme’s units, the same structure and sequence were used: warm-up and mobilisation exercises at the beginning of the training sessions, followed by coordination exercises and motor–cognitive games, strength and endurance training (with/without cognitive exercises), and finally, stretching and relaxation exercises. Balance as part of the coordination skills is a component of many exercises in the programme (e.g., exercises that involve a one-legged stance) and is additionally promoted by the outdoor setting itself (e.g., unstable surfaces). The structure of the individual units followed the F.I.T.T. principles (F = frequency, I = intensity, T = time, T = type) [67]. The individual exercises were low-threshold exercises that can easily be integrated into daily exercise routines and adapted to each person’s individual load (see Figure A1 for exercise examples). The exercise programme was delivered on-site by one out of three professional exercise instructors (M.Sc. sports scientists with a focus on prevention and rehabilitation and with exercise instructor licences for chronic diseases, e.g., heart or orthopaedic diseases, which were coached to implement the exercises of the intervention programme) in a group setting with a maximum of 15 participants per group and took place in outdoor public spaces, e.g., parks or green spaces in the respective city districts. Participants were instructed to wear appropriate clothing (e.g., sturdy shoes, dress in layers). As the programme took place in the spring and summer, participants were advised to take precautions in certain weather conditions (e.g., seek out shady spots and drink enough in the heat). Environmental infrastructure such as paved paths, park benches, lamp posts, or trees, as well as small training equipment such as elastic exercise bands, balls, a movement cube with individual mounting (e.g., cards for cognitive tasks), and chalk spray for markings on the ground were used in the programme. Examples of the progressive training principles used included the following: from simplicity to complexity, from stable to unstable support surfaces, from static to dynamic, and from exercises without to exercises with supplemental training material. The professional exercise instructors were informed in advance about the participants’ pre-existing conditions and adapted the individual exercises in terms of movement execution or intensity to each person’s health restrictions (e.g., limited joint mobility, pain, or circulatory distress). To control subjective as well as objective exertion during the exercise programme’s units, the participants’ heart rate and oxygen saturation were checked using a pulse oximeter (Orbisana GmbH, Augsburg, Germany). Their perceived exertion was assessed using a 5-step smiley scale [68] during different stages of the units (rest value, exertion value, recovery value). If necessary or in case of illnesses (e.g., cardiovascular disease), measurements with the pulse oximeter were conducted more frequently. Repeated checks of the participants’ physical condition during different phases of the exercise programme help train body awareness and teach participants how to detect their own physical stress limits, while these parameters were not used as outcomes. The exercise groups’ professional instructor took note of every participant’s attendance in each unit and promoted adherence verbally on site as well as via a text messaging app in a group chat. Reasons for absences did not have to be provided. The average attendance rate of analysed participants was 91.16% (±8.29%).

CG participants were instructed to continue to carry out their usual everyday activities during the entire study period. To control for physical activities, CG participants were asked about their involvement in exercise groups (41.1% participated in exercise groups) and the independent performance of exercises (58.9% independently performed exercises) during the whole test period at the follow-up measurement.

### 2.7. Outcome Measures and Analyses

#### 2.7.1. Procedure of Data Collection

For data collection, a coded ID number was used to pseudonymise participant information. The list of participants that were assigned to each group was only accessible to the project managers, who were also in charge of organising appointments for assessments and the intervention. Trained research assistants performed the assessments in compliance with a standardised measurement protocol.

Two measurements were carried out for both groups, which focused on demographic information as well as on the motor–cognitive exercise programme’s psychosocial, physical, and cognitive outcomes and feasibility: a baseline measurement time point (T1) before the start of the exercise programme (for IG) and at the beginning of the study period (for CG), as well as a follow-up measurement time point (T2) after the end of the programme (for IG) and 12 weeks after T1 (for CG). T1 was conducted at a time selected by the individual participants, and lasting about 1.5 h. Participants had received a letter by post in advance, which included general information on the study and a questionnaire. The letter also informed the participants of compensation in the form of an elastic exercise band with a clip and an illustrated exercise manual.

Most measurements were taken in the movement laboratories of the Chemnitz University of Technology. At the beginning, a survey of diseases using the Charlson Comorbidity Index [61] was conducted. Subsequently, the participants’ height (in cm, portable stadiometer by seca Deutschland, Hamburg, Germany) and weight (in kg, body fat scale by Tanita InnerScanV, Model BC-545 N, TANITA Corporation, Tokyo, Japan) were recorded. Physical and cognitive measurements were taken afterwards. At the end of T1, IG participants were informed about the goals, duration, place, time, and procedure of the outdoor motor–cognitive exercise programme.

Before T2, participants received a letter by post with an invitation to their measurement appointment and a questionnaire. The measurements were taken following the same procedure as in T1, using a different scheme that put physical and cognitive measurements in a different order for randomisation.

#### 2.7.2. Demographic, Feasibility, Psychosocial, Physical, and Cognitive Outcomes

A questionnaire was used to obtain information on the participants’ demographic situation, physical health, and social status. Additionally, at T2 and only for IG, subjective effects of and satisfaction with the outdoor motor–cognitive exercise programme were included into the questionnaire. Accordingly, statements about mobility, independent living and everyday activities, physical health and performance, psychological health and cognitive performance, aspects of pain and doctor’s visits, social and organisational aspects, as well as exercises and professional exercise instructors, were rated on a 5-point Likert scale (1 = not applicable; 2 = rather not applicable; 3 = partly applies; 4 = tends to apply; 5 = applies). Questions about the intention to independently continue with exercises and the intention to continue the motor–cognitive exercise programme in the exercise group were also added.

Furthermore, physical and cognitive functions were assessed using different, standardised, evidence-based, and valid instruments (see Table 2).

### 2.8. Statistical Analysis

There were missing values for every outcome parameter due to target group-related reasons (e.g., health issues with the upper or lower limbs during hand grip strength or 6MWT), practical measurement difficulties (e.g., missing information in the questionnaires), or personal reasons (e.g., reluctance to participate in a given measurement). The participants’ data analysed for every outcome parameter in both groups are presented in more detail in the results section (Table 3, Table 4, Table A1 and Table A2).

One-way ANCOVAs were used to conduct group comparisons (between IG and CG) at baseline as well as within-group comparisons of the T1 and T2 results. Further analyses to answer the first research question included ANCOVAs with repeated measures to determine time × group effects. To answer the second research question, both age and sex were used as covariates in all analyses. The post hoc analyses allowed for a more in-depth look at the ANCOVAs’ results in terms of age and sex within the two groups (IG and CG). A median split for the factor age was used for both groups: IG (younger group ≤ 71 years, *n* = 22 with 13 females; older group >71 years, *n* = 19 with 12 females) and CG (younger group ≤ 72 years, *n* = 30 with 23 female; older group > 72 years, *n* = 28 with 13 female). Feasibility outcomes of the motor–cognitive exercise programme (third research question) were descriptively analysed for IG. All statistical calculations were Bonferroni-corrected.

## 3. Results

### 3.1. Functional Performance of the Lower Limbs

**Figure 2:**
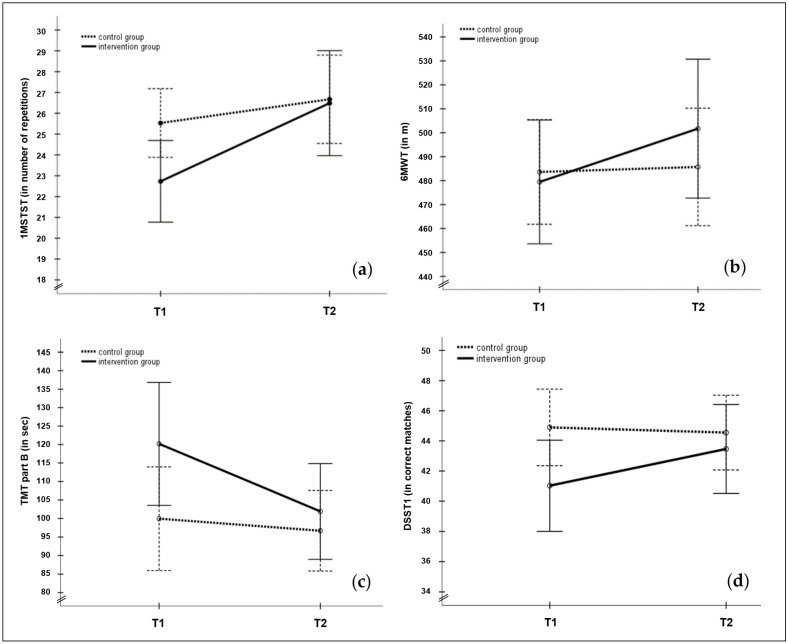
(**a**–**d**). Mean and SD for the outcome parameter 1-Minute-Sit-to-Stand-Test/1MSTST (**a**); 6-Minute-Walking-Test/6MWT (**b**); Trail Making Test B/TMT B (**c**); and Digit Symbol Substitution Test/DSST1 (**d**) for intervention group (IG: continuous line) and control group (CG: dashed line) in comparison to baseline (T1) and follow-up (T2) measurement.

The functional performance of the participants’ lower limbs, measured by the 1MSTST, differed significantly between the two groups at baseline (F(1,95) = 4.712, *p* = 0.032, $\eta_{p}^{2}$ = 0.046). Members of the IG completed 2.80 repetitions less, on average, than members of the CG at T1 (see Table 3 and Figure 2a). An increase in repetitions over one minute was observed for both groups at T2, indicating a similar level of performance, but with a stronger improvement for the IG participants with 3.76 repetitions more (F(1,40) = 9.500, *p* = 0.004, $\eta_{p}^{2}$ = 0.192; CG: +1.14 repetitions, F(1,57) = 4.226, *p* = 0.044, $\eta_{p}^{2}$ = 0.069). Tendentially significant effects for the factors group as well as time × group interaction were found (see Table 3), while the factor age (F(1,95) = 8.223, *p* ≤ 0.001, $\eta_{p}^{2}$ = 0.307) was significant. Female participants in both groups showed a significant performance increase (IG: +3.72 repetitions, F(1,24) = 5.540, *p* = 0.024, $\eta_{p}^{2}$ = 0.124; CG: +1.80 repetitions, F(1,35) = 6.771, *p* = 0.012, $\eta_{p}^{2}$ = 0.108). Within the IG, the performance of younger as well as older participants consistently and significantly increased over time (younger: +3.77 repetitions; older: +3.74 repetitions), with younger participants showing a higher level of performance (see Table A2). Significant differences between younger and older participants were only found at T1 in the IG (F(1,39) = 6.161, *p* = 0.017, $\eta_{p}^{2}$ = 0.136). Post hoc tests of the CG revealed that the functional performance of their lower limbs only improved significantly among the younger participants, with 1.84 repetitions more from T1 to T2 (F(1,29) = 5.745, *p* = 0.020, $\eta_{p}^{2}$ = 0.093). No between-group differences in the CG were identified post hoc between the two measurement time points for the factor age. At T1, both younger and older CG participants completed slightly more repetitions than those of the IG, while at T2, the two age groups’ performances were similar.

### 3.2. Functional Exercise Capacity

Both the IG and CG achieved a similar level of performance for the functional exercise capacity at baseline with a walking distance of 479.47 m for the IG and 483.62 m for the CG within 6 min (6MWT; see Table 3). The performance of the IG members improved at the follow-up measurement with an average of 22.24 m (F(1,39) = 2.193, *p* = 0.147, $\eta_{p}^{2}$ = 0.053), while the CG’s performance improved by 2.10 m (F(1,55) = 0.099, *p* = 0.754, $\eta_{p}^{2}$ = 0.002; see Figure 2b). No significant time × group interaction effect was detected for the walking distance, while a significant effect of the factors age (F(1,92) = 12.069, *p* ≤ 0.001, $\eta_{p}^{2}$ = 0.116), sex (F(1,92) = 14.263, *p* ≤ 0.001, $\eta_{p}^{2}$ = 0.134) and tendentially time × sex interaction effect (see Table 3) was evident. The significance of the factor age could not be confirmed in post hoc tests, but the groups differed at a descriptive level: the younger participants of both groups achieved a higher walking distance in both measurements, with younger members of the IG surpassing the improvements in the younger CG participants (IG Δ T1–T2 = 22.83 m, F(1,21) = 1.239, *p* = 0.237, $\eta_{p}^{2}$ = 0.023; CG Δ T1–T2 = 7.23 m, F(1,28) = 0.598, *p* = 0.443, $\eta_{p}^{2}$ = 0.011). While the performance of older CG participants declined from T1 to T2 (−3.38 m; F(1,26) = 122, *p* = 0.728, $\eta_{p}^{2}$ = 0.002), the improvement of the IG’s older participants was nearly as high (+21.53 m; F(1,17) = 0.901, *p* = 0.349, $\eta_{p}^{2}$ = 0.023) as it was for the younger members of the IG (see Table A1). Post hoc tests of the factor sex revealed that improvements in performance were driven in particular by the male participants, while the female participants in both groups performed at nearly the same level as at T1. Comparisons of IG male participants (F(1,14) = 5.547, *p* = 0.024, $\eta_{p}^{2}$ = 0.127) and between sexes at T2 for both groups, IG (F(1,38) = 9.341, *p* = 0.004, $\eta_{p}^{2}$ = 0.197) and CG (F(1,54) = 4.553, *p* = 0.037, $\eta_{p}^{2}$ = 0.078), showed significant results.

### 3.3. Hand Grip Strength

A total of 95.1% of the IG and 93.1% of the CG members defined themselves as right-handed. No baseline difference in the dominant hand’s grip strength and no changes in performance were found over time among the two groups. Men showed higher hand strength than women, as did younger participants as opposed to older participants, regardless of group and time of measurement. Nonetheless, ANCOVA analyses revealed significant age, sex, and time × sex effects for hand grip strength (see Table 3). As shown in Table A2, the effects of age could not be confirmed in post hoc analyses, while significant differences between the sexes were found for both groups and at both measurement time points (see Table A1). However, no significant development over time was reported for the factor sex.

### 3.4. Neurocognitive Performance

Baseline comparisons between the IG and CG of TMT A and TMT B revealed tendencies of significance, while no baseline difference was found between the two groups for the B/A ratio (see Table 4). Within TMT A, the performance of members of the IG descriptively improved (−4.15 s; F(1,40) = 1.474, *p* = 0.232, $\eta_{p}^{2}$ = 0.036), while participants of the CG showed a decrease in the level of functioning (+1.78 s; F(1,57) = 0.507, *p* = 0.479, $\eta_{p}^{2}$ = 0.009). The post hoc test for the factor age revealed that this performance decrease for CG in particular was driven by a higher age (+7.3 s; F(1,27) = 4.369, *p* = 0.041, $\eta_{p}^{2}$ = 0.072). No significant time × group interaction effect was identified but the factor of time and the time × age interaction effect were tendentially significant (see Table 4). The significant sex effect of the ANCOVA (F(1,95) = 5.540, *p* = 0.021, $\eta_{p}^{2}$ = 0.055) revealed that the decrease in performance among the CG members was driven by the male participants, who required 12.23 s longer, on average, compared to their female counterparts (T1; F(1,56) = 8.699, *p* = 0.005, $\eta_{p}^{2}$ = 0.134), and 18.85 s in T2 (F(1,56) = 7.674, *p* = 0.008, $\eta_{p}^{2}$ = 0.121). No within comparison of sexes in either group was significant (see Table A1).

Similar results to TMT A were found for TMT B: at a descriptive level, as is clearly visible in Figure 2c, IG members’ performance improved significantly (−18.28 s; F(1,40) = 4.226, *p* = 0.046, $\eta_{p}^{2}$ = 0.096), whereas that of the CG participants only changed slightly (−3.25 s; F(1,57) = 0.776, *p* = 0.382, $\eta_{p}^{2}$ = 0.013). The ANCOVA results were not confirmed by a significant interaction effect, while the factors sex (F(1,95) = 5.609, *p* = 0.020, $\eta_{p}^{2}$ = 0.056) and age (F(1,95) = 5.683, *p* = 0.019, $\eta_{p}^{2}$ = 0.056) showed significance. The factor group indicated a tendency for significance (see Table 4). Male CG participants were responsible for these results, with a minor increase in performance from T1 to T2 (−1.00 s), while the performance of female members of the CG improved by 4.63 s (see Table A1). Differences between the sexes were only found for the CG participants (in T1: F(1,56) = 9.967, *p* = 0.003, $\eta_{p}^{2}$ = 0.151; in T2: F(1,56) = 10.315, *p* = 0.002, $\eta_{p}^{2}$ = 0.156). No within comparison of sexes in either group was significant for TMT B. The performance of both IG male (−16.42 s) and female participants (−19.46 s) improved. For the factor age in TMT B, significant differences were found between younger and older participants in both the IG and CG at T2 (IG: F(1,39) = 4.398, *p* = 0.043, $\eta_{p}^{2}$ = 0.101; CG: F(1,56) = 5.095, *p* = 0.028, $\eta_{p}^{2}$ = 0.083). Older CG participants were the only sub-group whose performance in TMT B did not improve (see Table A2). No baseline differences, interaction, or covariate significance were identified in the TMT B/A ratio analysis.

A tendentially significant difference between groups at baseline was evident for DSST1 (see Table 4). The mean number of correct matches within 90 s for the CG members was the same at T1 (44.90) and T2 (44.55; F(1,57) = 0.181, *p* = 0.672, $\eta_{p}^{2}$ = 0.003), while the IG participants’ performance improved significantly from T1 to T2 (M Δ T1–T2 = 2.44; F(1,40) = 11.669, *p* = 0.001, $\eta_{p}^{2}$ = 0.226), but remained below the CG’s overall level of performance (see Figure 2d). A significant time × group interaction effect (F(1,95) = 6.943, *p* = 0.010, $\eta_{p}^{2}$ = 0.068), a significant effect of the factor sex (F(1,95) = 4.861, *p* = 0.030, $\eta_{p}^{2}$ = 0.049), as well as tendentially significant effects of the factors group and age (see Table A2) were observed for this parameter. Further analyses, presented in Table A1, revealed significant improvements from T1 to T2 of male (M Δ T1–T2 male = 3.43, F(1,15) = 8.049, *p* = 0.007, $\eta_{p}^{2}$ = 0.171) as well as of female IG participants (M Δ T1–T2 female = 1.92, F(1,24) = 4.389, *p* = 0.043, $\eta_{p}^{2}$ = 0.101). Additionally, a significant difference between sexes among the CG participants at T1 (F(1,56) = 7.669, *p* = 0.008, $\eta_{p}^{2}$ = 0.120) and T2 (F(1,56) = 9.011, *p* = 0.004, $\eta_{p}^{2}$ = 0.139) was found, with female participants achieving 7.45 (T1) and 7.19 (T2) more correct matches than their male counterparts (see Table A1). Younger participants of the IG showed a significant performance increase (+2.82 correct matches; F(1,21) = 8.217, *p* = 0.007, $\eta_{p}^{2}$ = 0.174), while for the CG, a significant T1 difference was found between age groups (younger: 47.93 correct matches; older: 41.46 correct matches; F(1,56) = 5.612, *p* = 0.021, $\eta_{p}^{2}$ = 0.091).

### 3.5. Memory

The baseline performance of memory among the members of both the IG and CG, measured by the DSST2 (F(1,95) = 5.424, *p* = 0.022, $\eta_{p}^{2}$ = 0.053), differed significantly. IG participants had 36.33% correct matches, while members of the CG achieved 47.89% correct matches, i.e., they matched nearly half of the symbols to the correct number. Both groups showed improved results at T2, but a significant change was detected for members of the IG (IG: 47.67% correct matches, (F(1,40) = 8.235, *p* = 0.007, $\eta_{p}^{2}$ = 0.171); CG: 51.00% correct matches, (F(1,57) = 2.036, *p* = 0.159, $\eta_{p}^{2}$ = 0.034). As presented in Table A1, female participants of the IG contributed to this result, showing a significant increase from 37.78% (T1) to 51.11% (T2) in correct matches (F(1,24) = 6.783, *p* = 0.013, $\eta_{p}^{2}$ = 0.148). In the CG, the male participants revealed significantly increased performance (T1: 41.44% correct matches; T2: 49.44% correct matches; F(1,21) = 5.600, *p* = 0.021, $\eta_{p}^{2}$ = 0.091), while female CG participants maintained the same high level of 51.89% correct matches. Only the older participants of the IG significantly improved their performance at T2, with a significant increase of +13.44% correct matches (see Table A2; F(1,18) = 5.227, *p* = 0.028, $\eta_{p}^{2}$ = 0.118). The ANCOVAs revealed no significant effects, but the factors group and time × group interaction showed tendencies for significance (see Table 4).

### 3.6. Feasibility

**Figure 3:**
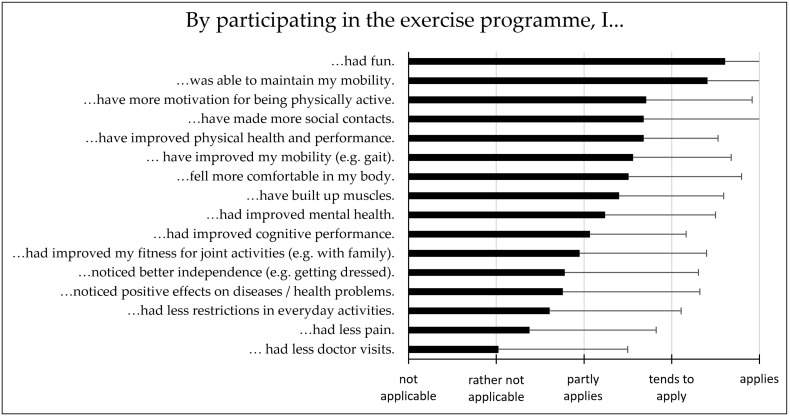
Subjective effects on biopsychosocial parameter by participating at the 12-week outdoor motor–cognitive exercise programme; evaluated by a 5-point Likert scale (1 = not applicable, 2 = rather not applicable, 3 = partly applies, 4 = tends to apply, 5 = applies).

As presented in Figure 3, the top five statements of subjective effects concerning the biopsychosocial parameter of the motor–cognitive exercise programme rated from 1 (“not applicable”) to 5 (“applies”) were “I had fun”. (M = 4.61 ± 0.86), followed by “I was able to maintain the good mobility I already had”. (M = 4.41 ± 0.86), “I have more motivation for being physically active”. (M = 3.71 ± 1.21), “I was able to socialise with other older people from my neighbourhood and foster a sense of community”. (M = 3.81 ± 1.45), and “I improved my physical health and performance” (M = 3.68 ± 0.85). Statements that were not applicable for most participants were as follows: “I had fewer visits to doctors or medical practitioners”. (M = 2.02 ± 1.46), “I was able to counteract my pain, if any”. (M = 2.38 ± 1.44), and “I have fewer restrictions in everyday activities, e.g., getting up from a chair”. (M = 2.61 ± 1.50).

Overall, participants were satisfied with the different aspects of the motor–cognitive exercise programme: the number of exercise units (M = 4.71 ± 0.60), length of exercise units (M = 4.85 ± 0.36), the number of participants (M = 4.93 ± 0.26), the accessibility of the location (M = 4.85 ± 0.42), the exercises in the programme (M = 4.20 ± 0.47), and the professional exercise instructor of the programme (M = 4.84 ± 0.36). 55.0% of the IG participants strongly plan to continue with exercises independently (e.g., with the elastic exercise band with a clip and the illustrated exercise manual received as compensation for study participation). Furthermore, 73.2% of participants (*n* = 30) strongly plan to continue exercising within their respective exercise groups in the outdoor setting, while 17.0% of the participants (*n* = 7) are still unsure about further group participation. Only 4 subjects (9.8%) stated that they no longer wished to participate in the groups.

## 4. Discussion

This study investigated the impact of a 12-week low-threshold outdoor motor–cognitive exercise programme on the psychosocial, physical, and cognitive health of OA. Furthermore, sex and age as influencing relevant confounders on health in older age were considered in detail. Motor and cognitive variables as targeted core elements of the exercise programme showed an improvement, evident especially in the functional performance of the IG and CG participants’ lower limbs, their functional exercise capacity, and their neurocognitive performance. Improvements were more pronounced in the members of the IG than of the CG, but, with the exception of the variable neurocognitive performance measured by the DSST1, they failed to show significant (interaction) effects. Different exercises as part of the intervention, such as squatting while naming words out of the same category (e.g., holiday destinations, favourite dishes, and girls’ names), alternate counting backwards in steps of three during the practice of different gaits, and execution of memorised movements in response to visual (e.g., colours, shapes, and numbers) or acoustic (e.g., beginning or stopping of music, names) signals, may have contributed to this result. Tendentially significant results were evident for many outcome parameters (e.g., functional performance of the lower limbs, memory). Age- and sex-related differences were revealed for certain parameters, showing that younger, male participants of the IG seemed to benefit most from the motor–cognitive exercise programme. For example, male IG participants significantly improved in their functional exercise capacity and neurocognitive performance, while younger IG participants showed a significant improvement in their functional performance of the lower limbs.

Despite an increase of about three repetitions from baseline to follow-up and a tendentially significant result of the time-to-group interaction in the 1MSTST, members of the IG showed low body strength performance at around the 25th percentile level [75]. CG participants were able to maintain their stable level of performance from around the 25th to 50th percentile level for both measurements. Many of the motor–cognitive exercises in the programme were explicitly designed for the muscles in the lower limbs. The motion sequence carried out in the 1MSTST was executed in several units using park benches. Despite practicing and a significant increase in repetitions, members of the IG could not regain the significant difference in baseline performance compared to the CG. A comparison with other findings for this test is difficult due to varying test times (5×, 30 s, 60 s), intervention design as well as age and health status. Compared with a significant improvement in performance of six repetitions in 30 s over 12 weeks [76], our results are considerably worse and not matchable. Other results of younger participants (median age: 67 years) with mild cognitive impairment are comparable to the performance of the CG participants in our sample [77]. A significant increase in performance was found among OA (85 years) and in a different setting (nursing home), but with a far weaker result than our sample [78]. This test illustrates the effect of age as well as the accompanying physical ageing processes, such as reduced number and size of muscle fibres and thus a decline in muscle strength, degenerative processes in the joints, and reduced bone density [79]. In contrast to other studies [75], all of our oldest participants (>80 years) were able to complete the test. Our results also confirm the significant impact of age, especially for members of the IG, with a performance difference between younger and older participants that remained stable over time, and an improvement reported for both age groups.

For the physical performance in 6MWT, the minimal clinically important difference (i.e., the smallest change in a treatment result) of +30 m has been defined by Holland et al. [80]. The IG participants narrowly missed this target with an increase of 22 m between T1 and T2. Although the walking distance of the CG participants improved by only 2 m, no significant time-to-group interaction effect was recorded. Walking (at different speeds and variety in gait) was part of our programme’s warm-up exercises, as were motor–cognitive games and endurance exercises. Walking at faster speeds more often within the exercise units may have been more useful for the outcome in 6MWT as well as for the participants’ everyday functioning (e.g., catching the bus), but this appeared to be a less safe option for the target group. Sex was confirmed to be significantly associated with performance in 6MWT [81]. In our study, the male participants of the IG benefited particularly from the intervention and achieved a clinically relevant and, at the same time, significant result with a difference of +56 m. Moreover, the male IG participants achieved the highest share of met targets (T1: 80.0%; T2: 75.0%) when applying the formula developed by Enright and Sherill [82] together with the female CG participants (T1: 86.1%; T2: 80.0%). Compared with slightly younger participants (66–70 years) in other studies that investigated the effects of multidimensional exercise programmes in healthy OA with similar programme durations [83,84,85], we find that the individuals in our sample walked shorter distances in general and showed a lower increase from baseline to follow-up. The influence of medication and comorbidities was not considered in this study and could be one reason for this difference in performance [80]. Conducting this test in an outdoor setting should not be a reason for the difference in performance since such tests in an indoor vs. an outdoor setting have been shown to be comparable [86]. However, small samples, high standard deviations, and the pilot character of this project may have influenced the results and its effects and should therefore also be considered when interpreting our findings. Nevertheless, our programme contributed to the maintenance of important everyday functions among OA, which is a major success for this age group. We recommend future motor–cognitive exercise programmes to include more speed walking exercises within a supervised setting and homogenise groups in terms of age and (orthopaedic/cardiovascular) comorbidities.

Amongst others, slow walking speed as well as muscle weakness measured by hand grip strength serve as indicators for functional limiting syndrome of frailty in OAs [87]. Additionally, hand grip strength is a quick, valid, and reliable measure for seniors. These are the reasons why we included this measurement in our study. IG participants’ walking speed improved while their hand grip strength decreased by −0.56 kg, which can be considered as maintaining performance at the functional level. Our results are not surprising because our motor–cognitive exercise programme did not include hand weights in the exercise equipment and did not focus on exercises that involve fine motor skills or other hand-strengthening movements. It should be noted that the maintenance of function in the target group, OA, represents a good result, is often the main goal of health promotion measures over time, and is crucial for healthy ageing [8]. Moreover, the sex and age differences we found in our sample for hand grip strength are comparable with the results of other studies involving OA [88].

Compared with the norm values for TMT B established by Tombaugh [89] (respective age groups averaged over years of education), the IG participants’ value was 22.08 s slower at the baseline measurement, with their performance improving significantly by 18.28 s at the follow-up measurement, which is 3.08 s slower than the norm. The CG already showed a faster execution time at T1 (−15.70 s than the norm) and was even able to improve its performance at T2 (−18.95 s). The same dimensions apply for the comparison of TMT A norms for the IG (T1: +10.72; T2: +6.57) and CG (T1: −29.93; T2: −28.15). Thus, the IG participants’ neurocognitive performance measured by TMT B was able to match that of the CG participants but was still above the norm values in both parts of the TMT. The highly inferior results of the CG compared to the norm are attributable to the difference in age group of the IG (age group 70–74) and the CG (age group 75–79). A high standard deviation was found in particular in the TMT B results of the IG at T1. In contrast to other studies, we did not set a time limit of 300 s for this test. Two members of the IG nearly exceeded this limit, while one participant’s performance was above the limit. Overall, 19 IG participants achieved a completion time of between 100 s and 200 s. The apparently challenging test for participants of the IG may be associated with the significant baseline group differences in cognitive health measured by the MoCA: the CG participants were classified as cognitively healthy on average (26.64 points), while the average IG participants showed a mild cognitive impairment (25.66 points). Despite the missing time-to-group interaction effect for both parts of the TMT, our findings are in line with those of other studies, including dual-task training interventions for OA [90,91,92]. Many exercises included in the motor–cognitive exercise programme were demanding in terms of motor speed and visual search, which are the functions needed for TMT B [93]. This could be a reason why IG participants show higher improvements in TMT B than in TMT A and a better improvement in TMT B compared to CG participants. In contrast to the existing literature, e.g., [89], sex had an impact on the performances in TMT A and B in our study. The female participants in both groups (CG > IG) showed a significantly better performance than the groups’ male members. This may partially be explained by the fact that within the CG, male participants had a higher mean age (77.55 ± 7.10 years) than their female counterparts (74.47 ± 7.95 years), and age has a high impact on neurocognitive performance. Additionally, the differences in hormonal changes between the sexes may have an influence on the differences in cognitive decline [94] and could therefore be an explanation for the female participants’ superiority in this cognitive task. The results of the TMT B/A ratio score, contrary to the individual results, indicated no difference in the groups or over time.

For the DSST1, the cognitive test that entails most different cognitive functions (e.g., attention, visual spatial skills, processing speed, memory, working memory, set shifting), a significant time-to-group interaction effect and a tendentially significant effect of group were found, attributable to the significant improvements (+2.4 symbols) in the IG. Despite our relatively small sample, it seems to be sufficient for the interaction in DSST1, complying with the specifications of the G*Power calculations. Furthermore, the factor of age has a significant effect. Again, the female members of the CG achieved a more superior and stable performance (+7–8 symbols) than their male counterparts, whereas the performance of the male and female participants in the IG was evenly balanced with significant improvements in both groups. Royer [95] already found that women performed better in this task than men with the same symbol set due to their better verbal encoding ability. The dimension of our results is comparable to those of a 10-week physically and mentally challenging instability free-weight resistance training with healthy German OA [96], even though a larger increase in symbol-number matches (+ seven symbols) was observed in the IG. A higher improvement in DSST1 performance (+ six symbols) was also reported in another study involving a 10-week multi-component intervention programme of habitual physical activity and cognitive function among older Korean adults with a mild cognitive impairment [97]. 

A significant baseline difference was found for the memory performance of the same task. Memory training was included in several units of our motor–cognitive exercise programme, and after 12 weeks, memory slightly increased for both groups. A significant improvement was only observed for the IG participants, while the time-to-group interaction effect narrowly missed the significance threshold. Various studies have shown that memory functions in community-dwelling OAs can be positively influenced by 12-week multicomponent exercise programmes that include cognitive training, e.g., [76,98,99]. It can be assumed that the baseline difference between the groups resulted in a lack of improvement in the memory performance of the individuals who were physically and cognitively trained.

Despite inconsistencies in the reporting of training protocols, programmes that include physical–cognitive training or combined exercise training have shown to improve both the motor and cognitive skills of participants [100]. In defiance of existing findings cf. [28,29,101], no evidence-based guidelines for outdoor exercise programmes for OA are currently available. Our results highlight the importance of developing tailored exercise programmes adapted to the needs and individual goals of OA (e.g., exercise loads depending on multimorbidity). At the purely descriptive level, males and younger IG participants benefitted the most from our motor–cognitive exercise programme in terms of physical and cognitive outcomes. Future interventions should implement such programmes in a more individualised, sex-sensitive way among groups that are divided according to their health limitations and in consideration of individual aspects of the ageing process. It is also possible that our 12-week intervention period was too short because health-related effects only occur after longer periods of regular exercise. For instance, it is recommended for older persons to engage in moderately intense mind–body training for 45 min to 60 min three times per week for at least six months to improve their working memory [101]. We will address this possible delay in health-related effects by repeating all of the above-listed parameters with the participants of both groups five to six months after T2 (second follow-up measurement/T3), and the results will be reported subsequently. This approach promotes the long-term implementation of our programme in a community setting. Other important health parameters, such as psychological or social health, were also surveyed in the context of this study and are still being evaluated. Overall, our study reinforces the feasibility of outdoor motor–cognitive exercise programmes that include low thresholds, are age-appropriate, take place within the close vicinity of the home, and are free of charge.

### Limitations

The results of our pilot study with a relatively small sample size are limited by the baseline differences in the group participants in terms of age, sex, general cognitive health, functional performance of the lower limbs and memory. The demographic factors of age and sex were considered in the statistical analyses. The life phase “old age” spans several decades, is subjected to a multidimensional process, and is highly individual [102]. With increasing age, individual differences in physical capacity and cognitive health parameters tend to rise, leading to a very heterogeneous group of OA, which was also the case in our sample. The range in age of the IG participants was 23 years, while that of the CG participants was 26 years, representing a typical German community with independently living OAs. Due to the individuality of the ageing process and the corresponding different manifestations of biopsychosocial health, the age range must be taken into account when interpreting the results and should be regarded as a limitation. However, it should be noted that this was a pilot and feasibility study under field research. The high age ranges, diversity of age, and other group characteristics could explain the lack of significant developments in some of the RCT groups’ variables over time. If data from further measurement points (T3) are available, multilevel analyses can be provided to estimate the within-person effects of physical activity. Such an approach can contribute to the individuality of the biopsychosocial reaction to physical training stimuli in OA to determine upper and lower limits and threshold values for health-promoting exercise activities in the future.

On the other hand, the differences in the sexes within groups nearly represent the city of Chemnitz distribution [2] as well as the preference of women to move and socialise in outdoor settings [103] to counteract their higher vulnerability to functional limitations [44] and therefore are neglectable. The groups’ cognitive and physical baseline levels varied. Furthermore, their sample size differed, as 15 IG participants were excluded from the analysis due to their low attendance rate. To be able to report on the effects of a relatively short 12-week exercise programme on individuals’ physical, cognitive, and psychosocial parameters, an attendance rate of at least 75% (18 out of 24 units) was required. This rate accounts for absenteeism of up to 3 weeks. In addition, we did not control for the activities the participants of the CG conducted throughout the intervention period. They were instructed to maintain their usual daily activities, which may have included physical or sporting activities, cognitive training, or others. The same applied to all of the (physical or sporting) activities of the participants in the IG carried out in addition to the bi-weekly exercise programme. Additionally, a large proportion of participants (CG > IG) were already regularly physically active before the start of the study (regularly exercising for at least 1–2 h per week: IG = 56.1%; CG = 75.4%) and this should be taken into account as limiting aspect. As regards the analyses, the often high standard deviations serve as a limiting factors for the explanatory power of our study results. The analysis was limited to group effects, which may be too highly aggregated to reflect intra-individual changes. Individual analyses would be more reliable in terms of determining which groups of persons benefitted most from the intervention.

Despite these limitations, the strengths of this study should also be emphasised: Even though this is a pilot study, a quasi-randomised controlled study design was conducted to detect the effects of such an outdoor motor–cognitive exercise programme. It is based on a holistic (bio–psycho–social) approach to promote healthy aging and the participants rated the feasibility of the exercise programme very well overall and enjoyed doing it. These factors can influence the participants’ long-term maintenance of physical activity positively.

## 5. Conclusions

The analysis of our outdoor motor–cognitive exercise programme reveals significant improvements in some physical (functional exercise capacity, functional capacity of the lower limbs) and all cognitive measures in the IG sample of OA. Neurocognitive performance, tested using the DSST1, showed a significant improvement over time with a medium effect size for the IG participants compared to the CG’s. Tendencies of a significant time-to-group interaction effect were evident for other parameters. Sex- and age-related differences for some of the physical and cognitive parameters were also found. We observed a high attendance rate during the 12 weeks of the exercise programme and the majority of IG participants stated that they wanted to continue exercising in their respective groups. Taking the positive results of the feasibility outcomes into account, our programme can be considered suitable and relevant for everyday life functions. It has been confirmed that the content-related orientation of the exercise-promoting intervention concept (needs-specific, group-based, free of charge, sustainable and transferable, socially and environmentally beneficial) is effective in terms of (cognitive and physical) health-related outcomes. Building on these results, our objective following the pilot phase of the study is to establish a motor–cognitive exercise programme that is a free-of-charge activity for OA in the community. The study made an exploratory contribution to improving exercise-related care for older people in urban areas. Therefore, the programme should be scaled up to other municipalities so that many OA can get access, and consequently benefit from the exercise programme’s health-related effects [65]. From a public health perspective, the participation of the target group and the inclusion of green space as a free exercise setting should be initiated for future health promotion interventions of OA by the community health authorities. Beyond that, every society or cultural space develops its own conception of aging and how to deal with health and its promotion through physical activity in old age. Therefore, exercise programmes such as ours will be particularly suitable for those societies and cultural areas that have a similar importance to physical activity in the context of healthy aging as in Germany. Implications of our study include the contribution to target group-specific further developments of municipal programmes in the area of health-promoting physical activity. Our exercise programme has been included in the offer of the city’s sports department and is thus receiving further municipal attention. The published manual [65] and feedback from participants for the further development of needs-specific interventions may contribute to future applications.

## Figures and Tables

**Table 1:** Participants’ characteristics and group comparisons using one-way ANOVA.

	Total (*n* = 99, 61 Female)		IG (*n* = 41, 25 Female)		CG (*n* = 58, 36 Female)		*F*	*p*	$\eta_{p}^{2}$
	M ± SD		M ± SD		M ± SD				
	Min	Max	Min	Max	Min	Max			
Age (years)	74.32 ± 7.28		72.46 ± 6.21		75.64 ± 7.73		4.748	0.032 *	0.047
	63	91	63	86	65	91			
BMI (kg/m^2^)	27.00 ± 3.73		27.26 ± 4.28		26.82 ± 3.32		0.332	0.566	0.003
	18.37	37.99	20.21	37.99	18.37	33.76			
ACCI	3.50 ± 1.35		3.17 ± 1.22		3.64 ± 1.33		3.155	0.079	0.032
	2	8	2	6	2	8			
SSES (ladder steps)	5.64 ± 1.29		5.46 ± 1.45		5.77 ± 1.15		1.377	0.243	0.014
	1	8	1	8	1	8			
MoCA (points)	26.23 ± 2.37		25.66 ± 2.54		26.64 ± 2.17		4.244	0.042 *	0.042
	21	30	21	30	21	30			

Note: IG = intervention group; CG = control group; BMI = body mass index calculated by weight/(height^2^); ACCI = age-adjusted Charlson Comorbidity Index [61]; SSES = subjective socio-economic status measured using the German version of the MacArthur scale (steps from 1–10) [62]; MoCA = Montreal Cognitive Assessment (points from 0–30) [60]; * *p* < 0.05.

**Table 2:** Outcomes and instruments used in the questionnaire, physical and cognitive measures.

Outcome		Instrument
Subjective socio-economic status (SSES)		MacArthur ladder scale (German version) [62] via questionnaire
Physical activity		Freiburg questionnaire for physical activity (“Freiburger Fragebogen für körperliche Aktivität“/FFkA) [59]
Functional performance of the lower limbs measured in completed repetitions within 1 minute		1-Minute-Sit-to-Stand-Test/1MSTST [69]
Functional exercise capacity measured in total walking distance in meters		6-Minute-Walking-Test/6MWT [70]
Hand grip strength measured in kilogramme (mean of three trials of the dominant hand)		Dynamometer by JAMAR (JAMAR R SmartHand Dynamometer, Performance Health Supply Inc, Cedarburg, USA) [71]
Neurocognitive performance (i.e., sustained attention, executive dysfunction, and visual exploratory capacity, processing speed, set shifting)	measured in time for completion in seconds	Trail Making Test/TMT A and B [72] + ratio of TMT B/A [73]
	measured in correct number–symbol matches within 90 seconds	Digit Symbol Substitution Test part 1/DSST1 [74]
Memory measured in correct number–symbol matches		Digit Symbol Substitution Test part 2/DSST2 [74]

**Table 3:** Outcome parameters of physical parameters for both groups at baseline (T1) and follow-up (T2) with the corresponding F-statistics.

**Functional performance of the lower limbs** (1MSTST; number of repetitions)										
	*n*	T1	*F*-statistics T1			T2	*F*-statistics T1–T2			
		M ± SD	*F*	*p*	$\eta_{p}^{2}$	M ± SD		*F*	*p*	$\eta_{p}^{2}$
**IG**	41	22.73 ± 5.22	4.712	0.032 *	0.046	26.49 ± 8.65	Time	1.148	0.287	0.012
**CG**	58	25.53 ± 7.00				26.67 ± 7.75	Group	3.028	0.085	0.031
							Sex	0.489	0.486	0.005
							Age	8.223	<0.001 **	0.307
							Time × Group	3.709	0.057	0.038
							Time × Sex	0.484	0.489	0.005
							Time × Age	0.637	0.427	0.007
**Functional exercise capacity** (6MWT; walking distance in m)										
	*n*	T1	*F*-statistics T1			T2	*F*-statistics T1–T2			
		M ± SD	*F*	*p*	$\eta_{p}^{2}$	M ± SD		*F*	*p*	$\eta_{p}^{2}$
**IG**	40	479.47 ± 95.62	0.072	0.789	0.001	501.71 ± 105.24	Time	0.383	0.537	0.004
**CG**	56	483.62 ± 71.52				485.72 ± 82.08	Group	0.156	0.694	0.002
							Sex	14.263	<0.001 **	0.134
							Age	12.069	<0.001 **	0.116
							Time × Group	1.612	0.207	0.017
							Time × Sex	3.663	0.059	0.038
							Time × Age	0.058	0.810	0.001
**Hand grip strength** (dominant hand; in kg)										
	*n*	T1	*F*-statistics T1			T2	*F*-statistics T1–T2			
		M ± SD	*F*	*p*	$\eta_{p}^{2}$	M ± SD		*F*	*p*	$\eta_{p}^{2}$
**IG**	41	27.98 ± 10.45	0.418	0.519	0.004	27.42 ± 9.88	Time	0.385	0.537	0.004
**CG**	57	26.70 ± 8.84				26.85 ± 7.67	Group	0.022	0.882	0.000
							Sex	152.422	<0.001 **	0.619
							Age	16.056	<0.001 **	0.146
							Time × Group	1.000	0.320	0.011
							Time × Sex	4.794	0.031 *	0.049
							Time × Age	0.098	0.755	0.001

Note: IG = intervention group; CG = control group; T1 = baseline measurement; T2 = follow-up measurement; 1MSTST = 1-Minute-Sit-to-Stand-Test; 6MWT = 6-Minute-Walking-Test; * *p* < 0.05; ** *p* < 0.01.

**Table 4:** Outcome parameters of neurocognitive parameters and memory for both groups at baseline (T1) and follow-up (T2) with the corresponding F-statistics.

**TMT A** (in s)										
	*n*	T1	*F*-statistics T1			T2	*F-*statistics T1–T2			
		M ± SD	*F*	*p*	$\eta_{p}^{2}$	M ± SD		*F*	*p*	$\eta_{p}^{2}$
**IG**	41	52.02 ± 20.51	2.803	0.097	0.028	47.87 ± 17.62	Time	3.687	0.058	0.037
**CG**	58	45.82 ± 16.33				47.60 ± 26.58	Group	1.354	0.247	0.014
							Sex	5.540	0.021 *	0.055
							Age	2.078	0.153	0.021
							Time × Group	0.985	0.323	0.010
							Time × Sex	0.358	0.551	0.004
							Time × Age	3.949	0.050	0.040
**TMT B** (in s)										
	*n*	T1	*F*-statistics T1			T2	*F*-statistics T1–T2			
		M ± SD	*F*	*p*	$\eta_{p}^{2}$	M ± SD		*F*	*p*	$\eta_{p}^{2}$
**IG**	41	120.19 ± 68.88	3.409	0.068	0.034	101.91 ± 38.69	Time	1.309	0.255	0.014
**CG**	58	99.95 ± 39.75				96.70 ± 43.68	Group	3.919	0.051	0.040
							Sex	5.609	0.020 *	0.056
							Age	5.683	0.019 *	0.056
							Time × Group	2.183	0.143	0.022
							Time × Sex	0.069	0.793	0.001
							Time × Age	0.937	0.335	0.010
**TMT B/A ratio**										
	*n*	T1	*F*-statistics T1			T2	*F*-statistics T1–T2			
		M ± SD	*F*	*p*	$\eta_{p}^{2}$	M ± SD		*F*	*p*	$\eta_{p}^{2}$
**IG**	41	2.47 ± 1.38	1.073	0.303	0.011	2.25 ± 0.87	Time	0.044	0.835	0.000
**CG**	58	2.25 ± 0.76				2.13 ± 0.62	Group	2.528	0.115	0.026
							Sex	0.142	0.707	0.001
							Age	2.913	0.091	0.030
							Time × Group	0.234	0.630	0.002
							Time × Sex	0.034	0.855	0.000
							Time × Age	0.134	0.716	0.001
**DSST1** (number of correct matches)										
	*n*	T1	*F*-statistics T1			T2	*F*-statistics T1–T2			
		M ± SD	*F*	*p*	$\eta_{p}^{2}$	M ± SD		*F*	*p*	$\eta_{p}^{2}$
**IG**	41	41.02 ± 8.56	3.789	0.054	0.038	43.46 ± 9.64	Time	0.818	0.368	0.009
**CG**	58	44.90 ± 10.51				44.55 ± 9.45	Group	2.783	0.099	0.028
							Sex	4.861	0.030 *	0.049
							Age	2.796	0.098	0.029
							Time × Group	6.943	0.010 *	0.068
							Time × Sex	0.219	0.641	0.002
							Time × Age	1.349	0.248	0.014
**Memory** (DSST2; number of correct matches, range: 0–9)										
	*n*	T1	*F*-statistics T1			T2	F-statistics T1–T2			
		M ± SD	*F*	*p*	$\eta_{p}^{2}$	M ± SD		*F*	*p*	$\eta_{p}^{2}$
**IG**	41	3.27 ± 1.90	5.424	0.022 *	0.053	4.29 ± 2.21	Time	0.097	0.756	0.001
**CG**	58	4.31 ± 2.38				4.59 ± 2.26	Group	2.986	0.097	0.030
							Sex	1.630	0.205	0.017
							Age	0.495	0.483	0.005
							Time × Group	3.717	0.057	0.038
							Time × Sex	0.359	0.551	0.004
							Time × Age	0.006	0.936	0.000

Note: IG = intervention group; CG = control group; T1 = baseline measurement; T2 = follow-up measurement; TMT = Trail Making Test; DSST = Digit Symbol Substitution Test; * *p* < 0.05.

## Data Availability

Data can be obtained from the corresponding author upon request.

## Appendix A

**Table A1:** Sex comparison of the outcome parameters.

**Functional performance of the lower limbs** (1MSTST; number of repetitions)									*F*-statistics within		
		*n*	T1 (M ± SD)			T2 (M ± SD)			*F*	*p*	$\eta_{p}^{2}$
**IG**	male	16	23.56 ± 4.89			27.38 ± 10.28			3.724	0.061	0.087
	female	25	22.20 ± 5.39			25.92 ± 7.60			5.540	0.024 *	0.124
	*F*-statistics between:		*F*	*p*	$\eta_{p}^{2}$	*F*	*p*	$\eta_{p}^{2}$			
			0.660	0.421	0.017	0.271	0.606	0.007			
**CG**	male	22	25.95 ± 5.78			26.00 ± 6.66			0.003	0.959	0.000
	female	36	25.28 ± 7.73			27.08 ± 8.42			6.771	0.012 *	0.108
	*F*-statistics between:		*F*	*p*	$\eta_{p}^{2}$	*F*	*p*	$\eta_{p}^{2}$			
			0.126	0.724	0.002	0.263	0.610	0.005			
**Functional exercise capacity** (6MWT; walking distance in m)									*F*-statistics within		
		*n*	T1 (M ± SD)			T2 (M ± SD)			*F*	*p*	$\eta_{p}^{2}$
**IG**	male	15	505.13 ± 100.87			561.30 ± 73.06			5.547	0.024 *	0.127
	female	25	464.07 ± 90.90			465.96 ± 106.45			0.000	0.919	0.000
	*F*-statistics between:		*F*	*p*	$\eta_{p}^{2}$	*F*	*p*	$\eta_{p}^{2}$			
			1.763	0.192	0.044	9.341	0.004 *	0.197			
**CG**	male	21	506.41 ± 64.17			515.01 ± 65.70			0.612	0.437	0.011
	female	35	469.94 ± 73.06			468.15 ± 86.68			0.044	0.835	0.001
	*F*-statistics between:		*F*	*p*	$\eta_{p}^{2}$	*F*	*p*	$\eta_{p}^{2}$			
			3.575	0.064	0.062	4.553	0.037 *	0.078			
**Hand grip strength** (dominant hand; in kg)									*F*-statistics within		
		*n*	T1 (M ± SD)			T2 (M ± SD)			*F*	*p*	$\eta_{p}^{2}$
**IG**	male	16	37.38 ± 8.78			36.08 ± 8.62			3.233	0.080	0.077
	female	25	21.96 ± 6.10			21.87 ± 5.86			0.001	0.881	0.001
	*F*-statistics between:		*F*	$\eta_{p}^{2}$	$\eta_{p}^{2}$	*F*	*p*	$\eta_{p}^{2}$			
			44.147	<0.001 **	0.531	39.584	<0.001 **	0.504			
**CG**	male	22	35.10 ± 7.47			34.28 ± 5.87			1.290	0.261	0.023
	female	35	21.42 ± 4.44			22.19 ± 4.19			1.824	0.182	0.032
	*F*-statistics between:		*F*	*p*	$\eta_{p}^{2}$	*F*	*p*	$\eta_{p}^{2}$			
			75.440	<0.001 **	0.578	82.401	<0.001 **	0.600			
**Neurocognitive performance: TMT A** (in s)									*F*-statistics within		
		*n*	T1 (M ± SD)			T2 (M ± SD)			*F*	*p*	$\eta_{p}^{2}$
**IG**	male	16	52.65 ± 21.00			47.97 ± 22.20			0.711	0.404	0.018
	female	25	51.63 ± 20.62			47.80 ± 14.48			0.742	0.394	0.019
	*F*-statistics between:		*F*	*p*	$\eta_{p}^{2}$	*F*	*p*	$\eta_{p}^{2}$			
			0.024	0.879	0.001	0.001	0.977	0.000			
**CG**	male	22	53.41 ± 22.14			59.30 ± 37.81			2.122	0.151	0.037
	female	36	41.18 ± 9.04			40.45 ± 12.40			0.053	0.819	0.001
	*F*-statistics between:		*F*	*p*	$\eta_{p}^{2}$	*F*	*p*	$\eta_{p}^{2}$			
			8.699	0.005 *	0.134	7.674	0.008 *	0.121			
**Neurocognitive performance: TMT B** (in s)									*F*-statistics within		
		*n*	T1 (M ± SD)			T2 (M ± SD)			*F*	*p*	$\eta_{p}^{2}$
**IG**	male	16	123.92 ± 89.47			107.50 ± 46.93			1.299	0.261	0.032
	female	25	117.79 ± 53.76			98.33 ± 32.92			2.851	0.099	0.068
	*F*-statistics between:		*F*	*p*	$\eta_{p}^{2}$	*F*	*p*	$\eta_{p}^{2}$			
			0.075	0.785	0.002	0.541	0.466	0.014			
**CG**	male	22	119.55 ± 45.88			118.55 ± 54.88			0.027	0.869	0.000
	female	36	87.98 ± 30.37			83.35 ± 28.58			0.962	0.331	0.017
	*F*-statistics between:		*F*	*p*	$\eta_{p}^{2}$	*F*	*p*	$\eta_{p}^{2}$			
			9.967	0.003 *	0.151	10.315	0.002 *	0.156			
**Neurocognitive performance: TMT B/A ratio**									*F*-statistics within		
		*n*	T1 (M ± SD)			T2 (M ± SD)			*F*	*p*	$\eta_{p}^{2}$
**IG**	male	16	2.44 ± 1.26			2.31 ± 0.68			0.100	0.754	0.003
	female	25	2.49 ± 1.48			2.21 ± 0.98			0.690	0.411	0.017
	*F*-statistics between:		*F*	*p*	$\eta_{p}^{2}$	*F*	*p*	$\eta_{p}^{2}$			
			0.009	0.924	0.000	0.134	0.716	0.003			
**CG**	male	22	2.38 ± 0.89			2.14 ± 0.63			2.086	0.154	0.036
	female	36	2.16 ± 0.67			2.12 ± 0.62			0.085	0.771	0.002
	*F*-statistics between:		*F*	*p*	$\eta_{p}^{2}$	*F*	*p*	$\eta_{p}^{2}$			
			1.159	0.286	0.020	0.010	0.923	0.000			
**Neurocognitive performance: DSST1** (number of correct matches)									*F*-statistics within		
		*n*	T1 (M ± SD)			T2 (M ± SD)			*F*	*p*	$\eta_{p}^{2}$
**IG**	male	16	40.31 ± 8.87			43.56 ± 10.77			8.049	0.007 *	0.171
	female	25	41.48 ± 8.51			43.40 ± 9.08			4.389	0.043 *	0.101
	*F*-statistics between:		*F*	*p*	$\eta_{p}^{2}$	*F*	*p*	$\eta_{p}^{2}$			
			0.178	0.676	0.005	0.003	0.959	0.000			
**CG**	male	22	40.27 ± 8.22			40.09 ± 9.60			0.019	0.892	0.000
	female	36	47.72 ± 10.84			47.28 ± 8.34			0.183	0.670	0.003
	*F*-statistics between:		*F*	*p*	$\eta_{p}^{2}$	*F*	*p*	$\eta_{p}^{2}$			
			7.669	0.008 *	0.120	9.011	0.004 *	0.139			
**Memory: DSST2** (number of correct matches)									*F*-statistics within		
		*n*	T1 (M ± SD)			T2 (M ± SD)			*F*	*p*	$\eta_{p}^{2}$
**IG**	male	16	3.06 ± 1.29			3.81 ± 1.91			1.969	0.200	0.042
	female	25	3.40 ± 2.22			4.60 ± 2.63			6.783	0.013 *	0.148
	*F*-statistics between:		*F*	*p*	$\eta_{p}^{2}$	*F*	*p*	$\eta_{p}^{2}$			
			0.303	0.585	0.008	1.252	0.270	0.031			
**CG**	male	22	3.73 ± 2.21			4.45 ± 2.44			5.600	0.021 *	0.091
	female	36	4.67 ± 2.44			4.67 ± 2.18			0.000	1.000	0.000
	*F*-statistics between:		*F*	*p*	$\eta_{p}^{2}$	*F*	*p*	$\eta_{p}^{2}$			
			2.174	0.146	0.037	0.118	0.732	0.002			

Note: IG = intervention group; CG = control group; T1 = baseline measurement; T2 = follow-up measurement; 1MSTST = 1-Minute-Sit-to-Stand-Test; 6MWT = 6-Minute-Walking-Test; TMT = Trail Making Test; DSST = Digit Symbol Substitution Test; * *p* < 0.05; ** *p* < 0.01.

**Table A2:** Age comparison of the outcome parameters.

**Functional performance of the lower limbs** (1MSTST; number of repetitions)									*F*-statistics within		
		*n*	T1 (M ± SD)			T2 (M ± SD)			*F*	*p*	$\eta_{p}^{2}$
**IG**	younger	22	24.50 ± 5.47			28.27 ± 10.13			5.014	0.031 *	0.114
	older	19	20.68 ± 4.16			24.42 ± 6.17			4.448	0.046 *	0.098
	*F*-statistics between		*F*	*p*	$\eta_{p}^{2}$	*F*	*p*	$\eta_{p}^{2}$			
			6.161	0.017 *	0.136	2.076	0.158	0.051			
**CG**	younger	30	26.63 ± 7.72			28.47 ± 8.48			5.745	0.020 *	0.093
	older	28	24.36 ± 6.07			24.75 ± 6.50			0.246	0.622	0.004
	*F*-statistics between		*F*	*p*	$\eta_{p}^{2}$	*F*	*p*	$\eta_{p}^{2}$			
			1.544	0.219	0.027	3.474	0.068	0.058			
**Functional exercise capacity** (6MWT; walking distance in m)									*F*-statistics within		
		*n*	T1 (M ± SD)			T2 (M ± SD)			*F*	*p*	$\eta_{p}^{2}$
**IG**	younger	22	505.02 ± 103.18			527.85 ± 123.38			1.239	0.273	0.032
	older	18	448.24 ± 77.12			469.77 ± 68.10			0.901	0.349	0.023
	*F*-statistics between		*F*	*p*	$\eta_{p}^{2}$	*F*	*p*	$\eta_{p}^{2}$			
			3.735	0.061	0.089	3.184	0.082	0.077			
**CG**	younger	29	492.87 ± 58.03			500.10 ± 64.18			0.598	0.443	0.011
	older	27	473.67 ± 83.63			470.29 ± 96.64			0.122	0.728	0.002
	*F*-statistics between		*F*	*p*	$\eta_{p}^{2}$	*F*	*p*	$\eta_{p}^{2}$			
			1.008	0.320	0.018	1.874	0.177	0.034			
**Hand grip strength** (dominant hand; in kg)									*F*-statistics within		
		*n*	T1 (M ± SD)			T2 (M ± SD)			*F*	*p*	$\eta_{p}^{2}$
**IG**	younger	22	29.81 ± 12.31			29.58 ± 11.19			0.136	0.715	0.003
	older	19	25.86 ± 7.57			24.91 ± 7.65			1.965	0.169	0.048
	*F*-statistics between		*F*	*p*	$\eta_{p}^{2}$	*F*	*p*	$\eta_{p}^{2}$			
			1.469	0.233	0.036	2.345	0.134	0.057			
**CG**	younger	29	27.06 ± 8.60			27.12 ± 7.50			0.008	0.928	0.000
	older	28	26.32 ± 9.21			26.58 ± 7.98			0.158	0.692	0.003
	*F*-statistics between		*F*	*p*	$\eta_{p}^{2}$	*F*	*p*	$\eta_{p}^{2}$			
			0.099	0.754	0.002	0.070	0.739	0.001			
**Neurocognitive performance: TMT A** (in s)									*F*-statistics within		
		*n*	T1 (M ± SD)			T2 (M ± SD)			*F*	*p*	$\eta_{p}^{2}$
**IG**	younger	22	48.12 ± 17.35			46.12 ± 13.61			0.181	0.673	0.005
	older	19	56.54 ± 23.31			49.90 ± 21.59			1.725	0.197	0.042
	*F*-statistics between		*F*	*p*	$\eta_{p}^{2}$	*F*	*p*	$\eta_{p}^{2}$			
			1.750	0.194	0.043	0.461	0.501	0.012			
**CG**	younger	30	44.94 ± 15.00			41.57 ± 14.00			0.994	0.323	0.017
	older	28	46.76 ± 17.88			54.06 ± 34.60			4.369	0.041 *	0.072
	*F*-statistics between		*F*	*p*	$\eta_{p}^{2}$	*F*	*p*	$\eta_{p}^{2}$			
			0.178	0.674	0.003	3.326	0.074	0.056			
**Neurocognitive performance: TMT B** (in s)									*F*-statistics within		
		*n*	T1 (M ± SD)			T2 (M ± SD)			*F*	*p*	$\eta_{p}^{2}$
**IG**	younger	22	109.41 ± 55.71			90.61 ± 30.88			2.341	0.134	0.057
	older	19	132.66 ± 81.34			115.00 ± 43.32			1.784	0.189	0.044
	*F*-statistics between		*F*	*p*	$\eta_{p}^{2}$	*F*	*p*	$\eta_{p}^{2}$			
			1.167	0.287	0.029	4.398	0.043 *	0.101			
**CG**	younger	30	90.94 ± 33.19			84.62 ± 33.84			1.509	0.224	0.026
	older	28	109.61 ± 44.34			109.64 ± 49.62			0.000	0.995	0.000
	*F*-statistics between		*F*	*p*	$\eta_{p}^{2}$	*F*	*p*	$\eta_{p}^{2}$			
			3.325	0.074	0.056	5.095	0.028 *	0.083			
**Neurocognitive performance: TMT B/A ratio**									*F*-statistics within		
		*n*	T1 (M ± SD)			T2 (M ± SD)			*F*	*p*	$\eta_{p}^{2}$
**IG**	younger	22	2.46 ± 1.51			2.07 ± 0.73			1.210	0.278	0.030
	older	19	2.49 ± 1.25			2.46 ± 0.98			0.004	0.984	0.000
	*F*-statistics between		*F*	*p*	$\eta_{p}^{2}$	*F*	*p*	$\eta_{p}^{2}$			
			0.004	0.949	0.000	2.147	0.151	0.052			
**CG**	younger	30	2.09 ± 0.67			2.09 ± 0.59			0.001	0.971	0.000
	older	28	2.41 ± 0.83			2.18 ± 0.66			2.491	0.120	0.043
	*F*-statistics between		*F*	*p*	$\eta_{p}^{2}$	*F*	*p*	$\eta_{p}^{2}$			
			2.677	0.107	0.046	0.320	0.574	0.006			
**Neurocognitive performance: DSST1** (number of correct matches)									*F*-statistics within		
		*n*	T1 (M ± SD)			T2 (M ± SD)			*F*	*p*	$\eta_{p}^{2}$
**IG**	younger	22	41.77 ± 7.09			44.59 ± 9.20			8.217	0.007 *	0.174
	older	19	40.16 ± 10.13			42.16 ± 10.23			3.574	0.066	0.084
	*F*-statistics between		*F*	*p*	$\eta_{p}^{2}$	*F*	*p*	$\eta_{p}^{2}$			
			0.357	0.553	0.009	0.643	0.427	0.016			
**CG**	younger	30	47.93 ± 11.97			46.40 ± 8.76			1.896	0.174	0.033
	older	28	41.64 ± 7.61			42.57 ± 9.91			0.649	0.424	0.011
	*F*-statistics between		*F*	*p*	$\eta_{p}^{2}$	*F*	*p*	$\eta_{p}^{2}$			
			5.612	0.021 *	0.091	2.438	0.124	0.042			
**Memory: DSST2** (number of correct matches)									*F*-statistics within		
		*n*	T1 (M ± SD)			T2 (M ± SD)			*F*	*p*	$\eta_{p}^{2}$
**IG**	younger	22	3.55 ± 2.02			4.41 ± 2.26			3.080	0.087	0.073
	older	19	2.95 ± 1.75			4.16 ± 2.19			5.227	0.028 *	0.118
	*F*-statistics between		*F*	*p*	$\eta_{p}^{2}$	*F*	*p*	$\eta_{p}^{2}$			
			1.013	0.320	0.025	0.129	0.721	0.003			
**CG**	younger	30	4.43 ± 2.60			4.67 ± 2.40			0.741	0.393	0.013
	older	28	4.18 ± 2.16			4.50 ± 2.15			1.312	0.257	0.023
	*F*-statistics between		*F*	*p*	$\eta_{p}^{2}$	*F*	*p*	$\eta_{p}^{2}$			
			0.164	0.687	0.003	0.077	0.782	0.001			

Note: IG = intervention group; CG = control group; T1 = baseline measurement; T2 = follow-up measurement; 1MSTST = 1-Minute-Sit-to-Stand-Test; 6MWT = 6-Minute-Walking-Test; TMT = Trail Making Test; DSST = Digit Symbol Substitution Test; * *p* < 0.05.
